# Pouch Revision in Combination with Placement of a MiniMizer Ring as a Revisional Procedure in Patient with Suboptimal Clinical Response or Recurrent Weight Gain After RYGB

**DOI:** 10.1007/s11695-025-07984-5

**Published:** 2025-07-05

**Authors:** Sophie C. van Helmond, Marijn T.F. Jense, Geert H.J.M. Verkoulen, Evelien De Witte, Pieter P.H.L. Broos, Jan Willem M. Greve, Evert-Jan G. Boerma

**Affiliations:** 1 Zuyderland Medisch Centrum, Sittard, Netherlands; 2 Maastricht University, Maastricht, Netherlands; 3 Nederlandse Obesitas Kliniek, Zeist, Netherlands

**Keywords:** RYGB, Pouch revision, MiniMizer

## Abstract

**Background and Objectives:**

Bariatric surgery, in particular Roux-en-Y gastric bypass (RYGB), has been a well proven and effective means of long-term management of severe obesity. However, about 10–35% of patients have a suboptimal clinical response (lowest quartile of weight loss after RYGB surgery) or suffer from recurrent weight gain often resulting in additional surgery. This study investigates the short-term results on weight loss and complications of surgical revision of the pouch in combination with placement of a MiniMizer ring in patients with a suboptimal clinical response or recurrent weight gain after RYGB.

**Methods:**

All patients who underwent pouch revision in combination with placement of a MiniMizer ring in the Zuyderland Medical Center between January 2016 and December 2021 were included. Weight, obesity-related complication resolution, and complications were monitored and reported during the follow-up appointments at the Nederlandse Obesitas Kliniek up to 2 years post-revisional surgery.

**Results:**

Thirty-six patients were included. These patients had a mean %TWL of 12.3 and 13.5 at 12 and 24 months post-revisional surgery. This resulted in a cumulative %TWL of 28.7 at 24 months after the revisional procedure. Patients with a superior response after the primary RYGB procedure (%TWL ≥ 35) responded better in %TWL post-revisional surgery compared to patients with a suboptimal response after the primary RYGB with a cumulative %TWL of 33.9 and 17.5 respectively. One ring-related complication occurred.

**Conclusions:**

Pouch revision in combination with placement of a MiniMizer ring appears to be a promising approach for managing patients with a suboptimal clinical response or recurrent weight gain after RYGB. The procedure offers significant additional weight loss with a low complication rate.

## Introduction

Roux-en-Y gastric bypass (RYGB) is one of the most frequently performed bariatric procedures worldwide [1]. RYGB has demonstrated successful short- and long-term weight loss results [2–4]. However, like with any other bariatric procedure, suboptimal clinical response or recurrent weight gain does occur in up to 35% of the patients 5–7 years post-RYGB. The underlying causes are multi-factorial. There can be both anatomical alterations, such as dilatation of the gastric pouch or dilatation of the gastro-jejunal anastomosis, Furthermore, an inability to adopt a healthy lifestyle as required after RYGB can surely be contributory [5–7]. When conservative measures such as improving dietary or exercise habits are not effective and when patients develop sub-optimal anatomic alterations, surgical revision should be considered.

Surgical modalities for revisional surgery as well as their mechanisms of action vary widely [8]. As the etiology of suboptimal clinical response or recurrent weight gain is very diverse and partly unknown, a standardized treatment protocol is lacking [9]. Within the Zuyderland Medical Center (ZMC) performing ring-augmented RYGB (raRYGB) is preferred over standard RYGB to optimize long term outcomes [10]. This is based on the concept that the ring prevents overeating and gastric pouch dilation, common reasons for suboptimal clinical response and/or recurrent weight gain after RYGB [6, 7]. The revisional procedure post-RYGB in the present study is targeted at these two pillars. A pouch resizing is performed to improve early satiety and to prevent overeating and a non-adjustable silastic ring is placed to enhance early satiety and to prevent secondary pouch dilatation.

This study aimed to determine the effect of pouch revision in combination with the placement of a MiniMizer ring in patients with a suboptimal clinical response or recurrent weight gain after RYGB on weight loss after 2 years of follow-up. Furthermore, predictors of outcome, such as prior weight loss results, were analyzed.

## Methods

### Study Design

This series includes all consecutive primary RYGB patients who underwent a pouch revision in combination with placement of a non-adjustable silastic ring (MiniMizer, Bariatric Solutions GmbH, Stein am Rhein, Switzerland) between January 2016 and December 2021 at ZMC. Data was prospectively collected and retrospectively analyzed. This study received local approval from the medical ethics committee in accordance with the ethical standards set forth in the 2013 Declaration of Helsinki.

### Participants and Eligibility Criteria

The inclusion criteria was comprised of previous RYGB patients who experienced either suboptimal clinical response or recurrent weight gain after primary RYGB surgery. A suboptimal clinical response was defined as patients who were in the lowest quartile of weight loss after RYGB surgery. Patients with recurrent weight gain were patients who initially achieved sufficient weight loss after RYGB surgery; however, they suffered from recurrent weight gain of > 10% of the lost weight. Excluded from this study were patients who had another indication for the surgical revision (e.g., reflux or obstruction) or were patients who underwent a ring-augmented or banded RYGB procedure (e.g., any other silicone ring or an adjustable gastric band) as the primary procedure.

### Treatment

In addition to the revisional, procedure all patients followed a multidisciplinary treatment plan at the Nederlandse Obesitas Kliniek (NOK) involving lifestyle modification. The multidisciplinary team consisted of a dietician, exercise therapist, psychologist, obesity physician, endocrinologist, and a bariatric surgeon.

### Surgical Procedure

**Fig. 1:**
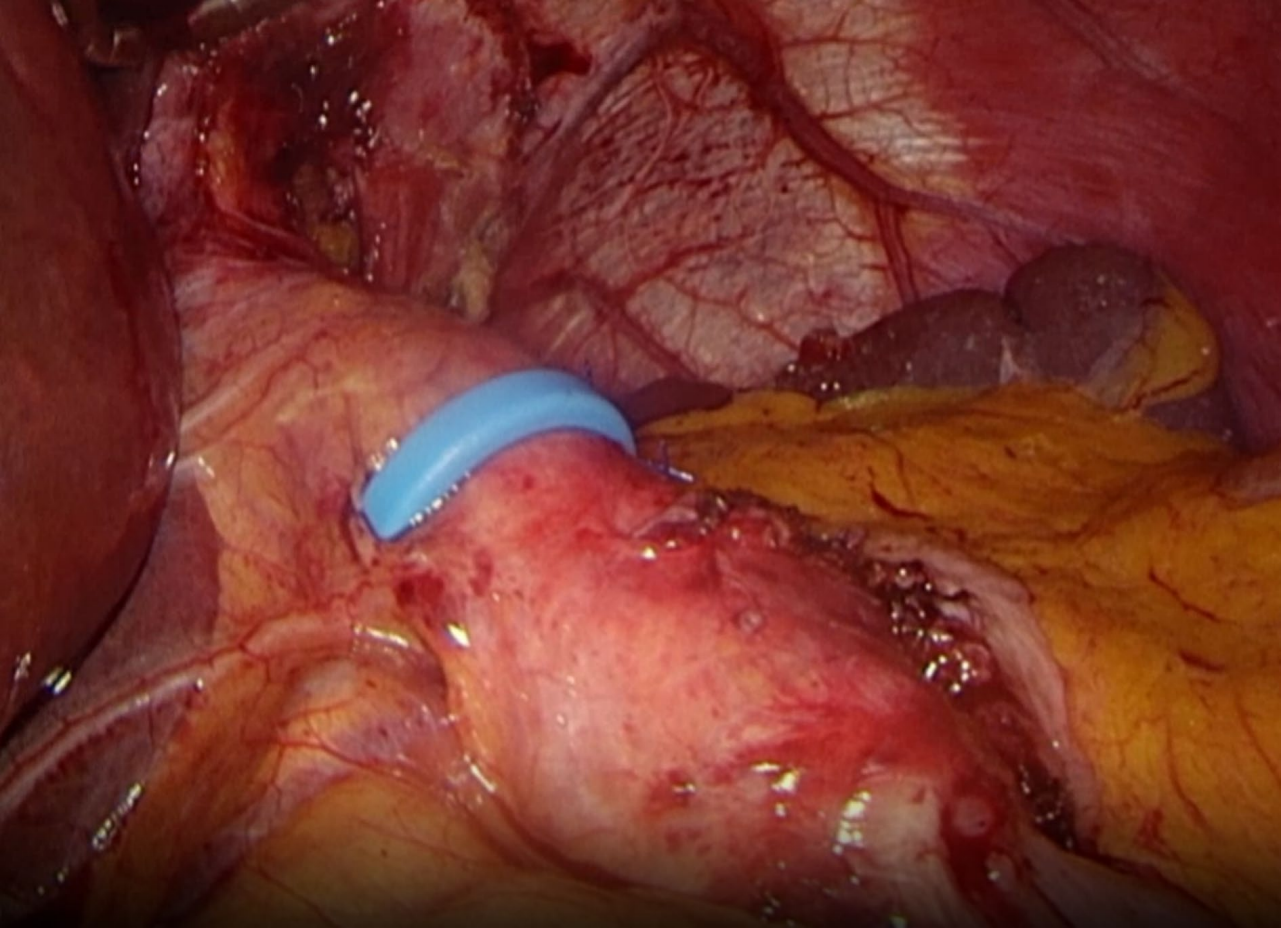
Intraoperative image presenting the MiniMizer ring placed on a revised gastric pouch

All included patients underwent a primary RYGB procedure. These primary procedures were performed in different hospitals; therefore, the surgical technique and limb lengths can differ between patients. All revisional operations were performed laparoscopically, using five trocars. Adhesions were taken down by sharp dissection either with endoscopic scissors or Harmonic (Ethicon, USA), the hiatus was checked for hiatal hernia and repaired by cruraplasty if needed, and the pouch was freed from adhesions to the remnant stomach. In every patient, the pouch was trimmed on a 40 French gastric bougie using a linear stapler with blue and gold cartridges of 60 mm (Echelon, Ethicon, USA). One should still be able to easily move the bougie through the pouch after pouch resizing. Therefore, during surgery, the bougie was moved in the pouch before staple firing. The MiniMizer (Bariatric Solutions GmbH, Stein am Rhein, Switzerland) was placed around the pouch with a circumference of 7.0 cm for females and 7.5 cm for males, 2–3 cm below the gastro-esophageal junction and at least > 2 cm above the gastro-jejunal anastomosis (Fig. 1). Before fixation of the ring, the gastric bougie was advanced through the hiatus, ring, and pouch to rule out any cause of obstruction. The ring was fixed with two non-absorbable sutures (Prolene 2.0, Ethicon, USA) to the lateral staple line of the pouch. One should be able to easily fit a 5-mm instrument between the ring and the pouch. If this is not possible, a larger circumference of the ring should be chosen. In none of the cases was a revision of the gastro-enterotomy performed. Nevertheless, if a “candy cane” was present, it was resected using a blue load of the linear stapler. Any sites of potential internal herniation were assessed and closed if patent with v-lock sutures.

### Primary Outcomes

#### Weight Change

The weight was collected at the following time points: prior to the primary RYGB procedure, after the primary RYGB, at screening for the revisional surgery, operation day, 3, 6, 12, and 24 months post-revisional surgery. A range of 3 months was accepted around the 12 months’ time point and 6 months around the 24 months’ time points to minimize loss to follow-up numbers. The percentage total weight loss (%TWL) was calculated for every time point over the 24-month period after revision using the weight at the day of screening prior to revisional operation. The %TWL of the primary operation was calculated using the highest weight loss after primary operation and the initial weight prior to any bariatric surgery. The group was divided into two subgroups based on the %TWL after the primary RYGB operation. A TWL of 35% or higher resulted in a classification as superior weight loss. Patients with a TWL lower than 35% were classified as suboptimal. The cumulative %TWL was calculated using the weight before the primary RYGB. For comparison with older literature, the percentage excess weight loss (%EWL) was also calculated for every time point using the following formula: (baseline weight − follow up weight)/(baseline weight − ideal body weight) × 100. Ideal body weight was set at a BMI of 25 resulting in the following formula: weight (kg) = 25 × length (m)^2^.

#### Obesity-Related Complications

During screening and at all follow-up, moments the following obesity-related complications were monitored: obstructive sleep apnea syndrome (OSAS), dyslipidemia, type 2 diabetes mellitus, and hypertension. The presence of any obesity-related complications, improvement, and remission were classified according to the definitions as stated by ASMBS [11].

#### Complications

Complications were prospectively registered in the patient files and added to the Dutch national database. All complications with a Clavien-Dindo classifications of 3b or higher were collected and analyzed for the present study.

#### Loss to Follow-Up

After revisional surgery, the patients continue their standard follow-up protocol. The majority of patients had their weight and obesity-related complications examined at the follow-up appointments in the clinic. When a patient missed a follow-up appointment, the patient was contacted by telephone and e-mail. We requested they reschedule their appointment. If these additional contact attempts proved unsuccessful, data was considered “missing.”

#### Statistical Analysis

Statistical analysis was performed using the IBM SPSS Statistics for Mac, version 27. All results were first tested for normality using the Shapiro–Wilk test. Differences in TWL within the study population for specific time points (primary procedure, nadir weight after primary procedure, screening, 3 months, 6 months, 12 months, 24 months) were tested for significance using the Wilcoxon signed rank test. Differences in TWL between superior and suboptimal groups were tested for significance using the Mann–Whitney *U* test. A *p*-value of less than 0.05 was regarded as statistically significant.

## Results

### Baseline Characteristics

A total of 36 patients with a mean BMI of 39.4 kg/m^2^ (range 27.9–56.6) at screening for revision were included. Of the 36 patients, 7 (19.4%) were male and 29 (80.6%) female, with a mean age of 49 years (range 23–63) at time of revision. The indication for revisional surgery was recurrent weight gain in 33 patients (91.7%), suboptimal clinical response in 1 (2.8%) or both in 2 patients (5.6%). A mean gap of 9 years (range 2–33) was present between the primary RYGB and revisional surgery. The primary RYGB was performed as a laparoscopic procedure in the majority of patients (83.3%). A mean %TWL of 36.0% was achieved after the primary RYGB. On average, patients gained 26.1 kg (range 4.5–80.1) in weight (18.7 mean %TWL) before the revisional procedure.

### Weight Loss

**Fig. 2:**
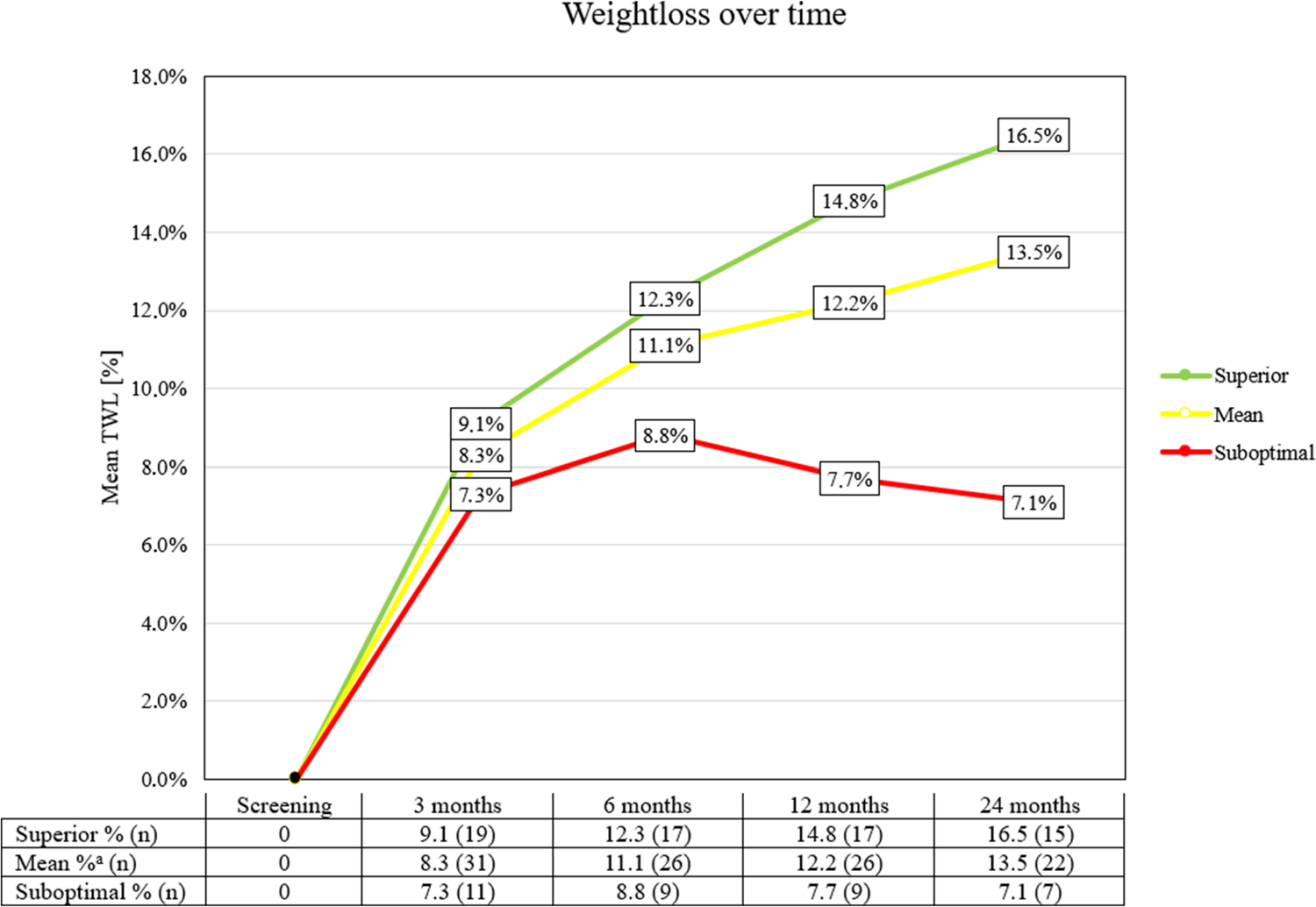
Weight loss post-revisional surgery. ^a^One patient had no post-RYGB weight and could therefore not be included in either the superior or suboptimal group

A %TWL of 12.2% (EWL% = 32.9) and 13.5% (EWL % = 48.0) was achieved at 12 months and 24 months after revisional surgery respectively (Fig. 2). The difference between mean %TWL at screening and 1- and 2-year follow-up points was statistically significant with a *p* < 0.001 at both time points. Superior weight loss patients (%TWL ≥ 35) achieved a %TWL of 16.5, and patients from the suboptimal group (%TWL < 35) achieved a %TWL of 7.1 at 24 months post-revisional surgery (Fig. 2). The differences among both groups were not statistically significant (*p* = 0.133 at 12 months, *p* = 0.202 at 24 months).

### Cumulative Weight Loss

**Fig. 3:**
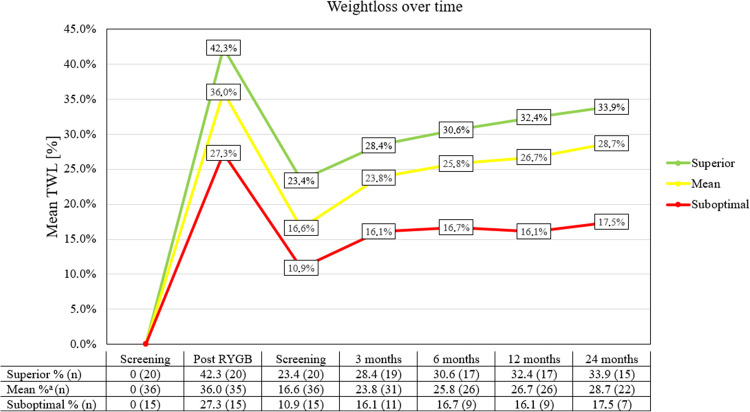
%Total weight loss from screening for primary procedure until 2 years after revisional procedure. ^a^One patient had no post-RYGB weight and could therefore not be included in either the superior or suboptimal group

A cumulative %TWL of 26.7% (EWL% = 56.9) and 28.7% (EWL% = 60.0) was achieved at 12 months and 24 months after revisional surgery respectively. Superior responders (%TWL ≥ 35) and suboptimal responders (%TWL < 35) achieved a cumulative %TWL of 33.9% and 17.5%, respectively, at 24 months post-revisional surgery (Fig. 3). Independent sample Mann–Whitney *U* testing was performed; the differences among both groups were statistically significant (*p* < 0.001 at 12 months, *p* = 0.045 at 24 months).

As an overview of the data, Table 1 was added, presenting %TWL, %EWL, and weight over all follow-up moments.

**Table 1:** Overview of weight results over time

	Screening	Post-RYGB	Screening	3 months	6 months	12 months	24 months
Total body weight (kg)	152.3	82.3	125.1	100.5	91.1	87	88.9
Total weight loss (%)	-	36	16.6	23.8	25.8	26.7	28.7
Excess weight loss (%)	-	78.8	36.1	51.4	55.4	56.9	60.0

### Obesity-Related Complications

Descriptives of obesity-related complications that were present at screening and post-revision are shown in Table 2. Since the number of obesity-related complications was rather low, no statistical testing could be performed on this data.

**Table 2:** Obesity-related complications remission post-revisional surgery

Obesity-related complications	Screening revision *n* = *36*	Remission post-revision^a^
*Hypertension*	8/34	2/3 (66%) + 1^b^
*DM*	2/34	0/0 (n.a.)
*Dyslipidemia*	5/34	0/2 (0%)
*OSAS*	2/34	0/1 (0%)

^a^Calculated as remission/number of obesity-related complications with data available at 24-month follow-up (percentage of remission)

^b^1 de novo diagnosis of hypertension occurred

### Complications

In this study group, there were no short-term complications (< 30 days postoperative) with a classification of Clavien Dindo 3b or higher, such as anastomotic leakage or bleeding. In terms of long-term complications, three patients suffered from dysphagia (8.3%). However, no organic explanation was appreciated on a barium swallow test. There were no cases of dysphagia for which the ring had to be removed. There was one ring-related complication which was scored as a Clavien Dindo 3b or higher: the patient required repositioning of the MiniMizer due to gastric prolapse through the ring (“slippage”). No ring erosion occurred in this study population. Additional surgery was performed in 4 patients: 3 patients underwent laparoscopic cholecystectomy for symptomatic cholecystolithiasis, and 1 patient underwent laparoscopic incisional hernia repair.

### Loss to Follow-Up

Per time point, different percentages of patients were lost to follow-up, ranging from 0 to 52.8% as presented in all figures and tables. After the 2-year follow-up, a total of 22 patients (61.1%), 15 of the superior responders and 7 of the suboptimal responders, were still being followed.

## Discussion

This study demonstrates that pouch revision in combination with placement of a MiniMizer ring results in significant additional weight loss in patients with a suboptimal clinical response or recurrent weight gain after RYGB.

### Weight Loss

Patients achieved a total %TWL of 13.5 (EWL = 48.0%) post-revisional surgery and a cumulative %TWL of 28.7 (EWL% = 60.0) post-RYGB. These results are promising when compared to existing literature on alternative revisional procedures after RYGB [7]. Some of the possible revisional procedures are distalization of the RYGB, sometimes combined with banding, pouch or gastro-jejunal anastomosis resizing, banding of the gastric pouch, endoscopic revision, and biliopancreatic diversion with duodenal switch. A meta analysis by Kermansaravi et al. concludes that conversion to a biliopancreatic diversion with duodenal switch results in the most effective results treating weight recurrence with an EWL of between 56.4 and 61.5% after 2 years. Although the weight loss differences are comparable to our technique, the number of serious long-term complications is expected to be lower with our technique. Studies focusing solely on pouch resizing or ring augmentation have reported varying outcomes. Al-Bader et al. reported an EWL% of 29.1 for a pouch resizing in patients with suboptimal clinical response at a mean follow-up of 14.1 months [12]. A more recent study by Boerboom et al., on non-adjustable ring placement (without pouch revision) as a revisional procedure, reported a cumulative %TWL of 26. Within this study, the principle of superior- and suboptimal responders after primary RYGB was introduced in which superior responders achieved a higher %TWL compared to suboptimal responders with a mean difference of 9.4% TWL after revisional procedure [13]. This finding suggests that initial response to RYGB may predict outcomes of revisional surgery. Franken et al. reported higher %TWL values of 21.2 and 17.0 at 12 and 36 months, respectively, for procedures where pouch revision was combined with a ring placement compared to solely revising the pouch. However, they also noted a higher rate of complications and re-interventions [14].

While some studies have not found a significant relationship between pouch size and primary non-response, the addition of the MiniMizer ring placement to pouch resizing is believed to prevent secondary pouch dilation [15, 16]. The hypothesis is that the ring prevents pouch dilation by reducing food intake. When patients do not chew their food adequately, large food particles may enter the pouch, and the presence of the ring can lead to a sensation of dysphagia. Therefore, patients are trained to chew their food thoroughly, which promotes smaller portion sizes and may consequently reduce the risk of pouch dilation. Besides, if one does not trim the pouch the risk of impression of the ring on the pouch is higher with the risk of dilatation and dysphagia as a consequence. The dual approach of ring augmentation and revision of the pouch may explain the favorable outcomes observed in the present study.

### Complications

The current study showed only one ring-related complication, which is 2.8% of this study population. The only ring-related complications was slippage of the ring, which could be resolved by repositioning. No erosion of the ring was seen in this study population. Dysphagia occurred in 3 patients (8.3%); however, as no explanation was found on barium swallow, it is questionable if these were ring related. In one patient, the complaints resolved after dietary advice.

A recent systematic review on revisional treatments for suboptimal clinical response and recurrent weight gain by Franken et al. reported a percentage of 13.3–14.3 ring (non-adjustable and adjustable) removals in ring-augmented revisional procedures (laparoscopic banding (LGB) and LGB combined with pouch resizing) [14]. In one of the studies by Moon et al., the use of a pericardial patch ring was thought not to be feasible, safe, or effective due to unsatisfying weight loss results over a period of 2 years and high complication rates (11% patch related complications) [17]. As the pericardial patch is a biological material and thus absorbable, this unsatisfactory result is to be expected. Felsenreich et al. reported dysphagia in 31% of their patients and a 17% ring removal rate with a ring circumference of 6.5 cm. The study by Boerboom et al. also reported a high complication rate of 23%, most often dysphagia, when using rings with a mean circumference of 6.8–7.1 cm [13]. The difference in complication rates between these studies and the current study can possibly be found in the position of the ring, the ring size, and the fact that no revision of the pouch was performed. The circumference and closing position of the non-adjustable ring of 7.0 and 7.5 cm and the loose but profound fixation of the MiniMizer around the pouch as performed in the current study result in a safe and effective procedure with minimal complications.

### Obesity-Related Complications

As patients received prior bariatric surgery, only a small number of obesity-related complications are present at the time of screening for the revisional procedure. As missing values during follow-up were frequent in this outcome variable, it is not possible to draw any conclusion based on these numbers, and no statistical tests could be performed on this data. A previous study by Giannopoulus et al. concluded no difference in obesity-related complication resolution between primary and revisional bariatric surgery, studying only RYGB and sleeve gastrectomy [18]. Although data on obesity-related complications is limited, Ryan et al. do present that a reduction in weight will eventually result in reduction of obesity-related complications [19], underlining the importance of the additional weight reduction which can be obtained with pouch revision and ring augmentation.

### Limitations of the Study

The present study has several limitations, including its retrospective nature and relatively small sample size. Besides, this study is a single-center and single-arm study with a high loss to follow-up rate (> 50% after 2 years) although comparable to other bariatric studies. Therefore, the results of this study should be interpreted with caution. However, to our knowledge, this is the first study reporting on the combination of pouch revision and MiniMizer placement as a revisional procedure for suboptimal clinical response of recurrent weight gain after RYGB. Future research should focus on larger, prospective studies, if possible multi-center, to explore the findings of this study. Additionally, long-term follow-up beyond 2 years would provide valuable insights into the durability of weight loss outcomes and potential long-term complications.

## Conclusion

Pouch revision in combination with placement of a MiniMizer ring appears to be a promising approach for managing patients with a suboptimal clinical response or recurrent weight gain after RYGB. The procedure offers significant additional weight loss with a low complication rate.

## Data Availability

No datasets were generated or analysed during the current study.
